# Heteropolyacid coupled with cyanoguanidine decorated magnetic chitosan as an efficient catalyst for the synthesis of pyranochromene derivatives

**DOI:** 10.1038/s41598-022-21196-2

**Published:** 2022-10-11

**Authors:** Golnaz Rahimzadeh, Mahmood Tajbakhsh, Mansoureh Daraie, Ali Ayati

**Affiliations:** 1 Department of Organic Chemistry, Faculty of Chemistry, University of Mazandaran, Babolsar, Iran; 2 Department of Chemistry, Science and Research Branch, Islamic Azad University, P.O. Box 14515/775, Tehran, Iran; 3 Department of Chemical Engineering, Quchan University Technology, Quchan, Iran

**Keywords:** Catalysis, Organic chemistry, Surface chemistry

## Abstract

In this study, a novel nanocatalyst was successfully prepared by heteropolyacid immobilization of magnetic chitosan-cyanoguanidine composite and fully characterized by different analysis methods, including FTIR, XRD, TGA, SEM, and EDS. The catalytic activity of fabricated composite was examined in a one-pot three-component reaction, involving the diverse active methylene compounds, various aryl aldehydes, and malononitrile in water. The results revealed the efficient catalytic performance of composite, while all reactions proceeded smoothly and led to the formation of the corresponding pyranochromene derivatives in high to excellent yields.

## Introduction

In recent years, the heteropolyacids (HPAs) have attracted great attention, due to their high catalytic activity, strong Brønsted acidity, low toxicity, and tunable redox properties^1,2^, which render them a numerous applications in all fields of chemistry, both academically and industrially^3^. The critical weakness of HPAs attributed to their high solubility in polar solvents and low surface area, which highly limited their catalytic performance and made some difficulty in their separation from the reaction mixture at the end of the reaction^4^. The dispersion of HPAs on the high surface area solid supports is an efficient strategy to circumvent these problems which led to increasing their active surface area and facilitating their separation from the reaction media to be re-used in another successive run^5,6^.

Despite achievements in promoting the catalytic potency of a wide variety of catalysts from different points of view, finding an easy and efficient separability of heterogeneous catalysts is still a challenge^7–10^. In this regard, the immobilization of HPAs catalyst on the magnetic supports is an efficient approach, in which the heterogeneous catalyst can be easily separated from the reaction mixture by an external magnet without encountering the inherent leaching problems^11^.

Chitosan is one of the most abundant and intriguing biopolymers in nature, which is widely used in environmental remediation, food, agricultural, and pharmaceutical industries^12,13^. It possesses intrinsic physicochemical features, such as biodegradability, biocompatibility, high chemical stability, high reactivity, and outstanding chelation behavior^14^. Considerable efforts have been devoted to preparing magnetic chitosan-based materials in recent years^15–17^ and extensively employed as catalyst supports in a wide range of organic reactions^18–20^. Despite this wide range of applications, there are a few reports on the exploiting of magnetic chitosan, as HPAs’ supports in the catalytic reactions^21,22^.

**Figure 1:**
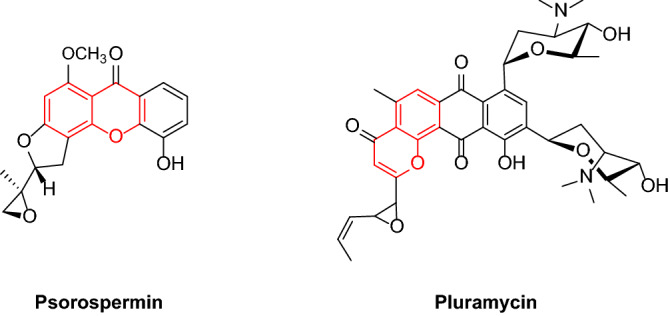
Some pharmacologically important chromene derivatives.

The chromene scaffold generates the nucleus of a class of naturally occurring compounds. It is also a part of pharmacophores of a wide variety of biologically active compounds^23^, including anticancer agents^24^. Several hetero- and carbo-annulated chromene compounds were found antifungal^25^, antiplatelet^26^, and also anticancer molecules^27^. Figure 1 shows the structures of some pharmacologically important chromene derivatives.

In our previous studies, the extraordinary performance of different kinds of HPAs catalysts in several organic reactions was studied^28–36^. Also, the application of nanoparticles^37–41^ and particularly magnetic Fe_3_O_4_^42–47^, as efficient and easily separable catalysts, was demonstrated in various organic reactions. In this work, a novel magnetically recoverable catalyst containing immobilized phosphotungstic acid (H_3_PW_12_O_40_, HPW), as a Keggin type HPA, cyanoguanidine modified magnetic chitosan was designed. The Al_2_O_3_/Fe_3_O_4_ core–shell nanoparticles were used as the magnetic core of composite, which improved its chemical stability and led to formation a super-paramagnetic catalyst that can be easily separated from the medium by an external magnet^48,49^. The phosphotungstic heteropolyacid embedded magnetic chitosan-cyanoguanidine (HPW@Fe_3_O_4_–Al_2_O_3_–CS–CG) catalyst was characterized using different methods and successfully applied in a one-pot three-component synthesis of pyaranochromene derivatives.

## Experimental

### Chemicals and instruments

The used chitosan in this work was purchased from Acros Organics, and the other chemicals, including phosphotungstic acid hydrate [H_3_(PW_12_O_40_) xH_2_O], iron (II, III) oxide Nano powder, aluminium isopropoxide (≥ 98%), glutaraldehyde solution (25%), cyanoguanidine, 3,4-methylene-dioxy-phenol, dimedone, 4-Hydroxy coumarine, 3-methyl-1-phenyl-pyrazole-5-one, malononitrile, and the used solvents, such as toluene, CH_3_CN, HOAC, and EtOH were all supplied from Sigma-Aldrich Co. and used without further purification.

The prepared HPW@Fe_3_O_4_–Al_2_O_3_–CS–CG catalyst was characterized by analyzing the obtained data. Fourier transform infrared (FT-IR) spectra were recorded by VERTEX-70 infrared spectrometer (by ATR method). The XRD measurement was carried out using a PANalytical Empyrean powder diffractometer using Cu Ka radiation in 2θ-range of 15–100° (step size = 0.0001°) under ambient temperature and pressure. To investigate the catalyst size and surface morphology Scanning electron microscope (SEM) images were taken using the ultra-high resolution Tescan MIRA 3 scanning electron microscope. Melting points were measured by an electrothermal 9200 apparatus via the capillary tube method and the TMS, as an internal standard (DMSO solution), was employed to record the ^1^H-NMR and ^13^C-NMR spectra on a Bruker AQS 500-AVANCE spectrometer at 500 and 125 MHz respectively. The reactions were monitored by TLC and all products were recognized by comparison of their physical and spectroscopic data with those of authentic samples in identical.

### Preparation of catalyst

#### *Synthesis of magnetic Fe*_*3*_*O*_*4*_–*Al*_*2*_*O*_*3*_–*CS*

Above all, the iron oxide nanoparticles/Al_2_O_3_ core–shell nanospheres were prepared according to the reported method by Tanhaei et al.^16^. To prepare Fe_3_O_4_–Al_2_O_3_–CS, 2 g of chitosan was dissolved in 100 mL of acetic acid and stirred for 2 h to be dissolved and homogenized. Then, the as-prepared magnetic Fe_3_O_4_/Al_2_O_3_ particles were dispersed in the chitosan solution in an ultrasonic bath. Next, the glutaraldehyde solution (25 wt%) was added until a brown gel product was formed. It was washed several times with acetic acid solution (2%) and deionized water. The obtained *Fe*_*3*_*O*_*4*_–*Al*_*2*_*O*_*3*_–*CS* solid was finally separated using an external magnet and dried at 60 °C overnight^50^.

#### *Synthesis of Fe*_*3*_*O*_*4*_–*Al*_*2*_*O*_*3*_*–CS–CG*

Initially, 2 g of Fe_3_O_4_–Al_2_O_3_–CS was grounded and dispersed in 100 mL of hydrochloric acid solution 1% (v/v), followed by addition of 1.06 g cyanoguanidine under magnetic agitation. The mixture was stirred for 4 h at 90 °C, washed three times with water, and then cooled at room temperature to obtain the cyanoguanidine modified Fe_3_O_4_–Al_2_O_3_–CS.

#### *Immobilization of H*_*3*_*PW*_*12*_*O*_*40*_* onto Fe*_*3*_*O*_*4*_–*Al*_*2*_*O*_*3*_–*CS*–*CG*

**Figure 2:**
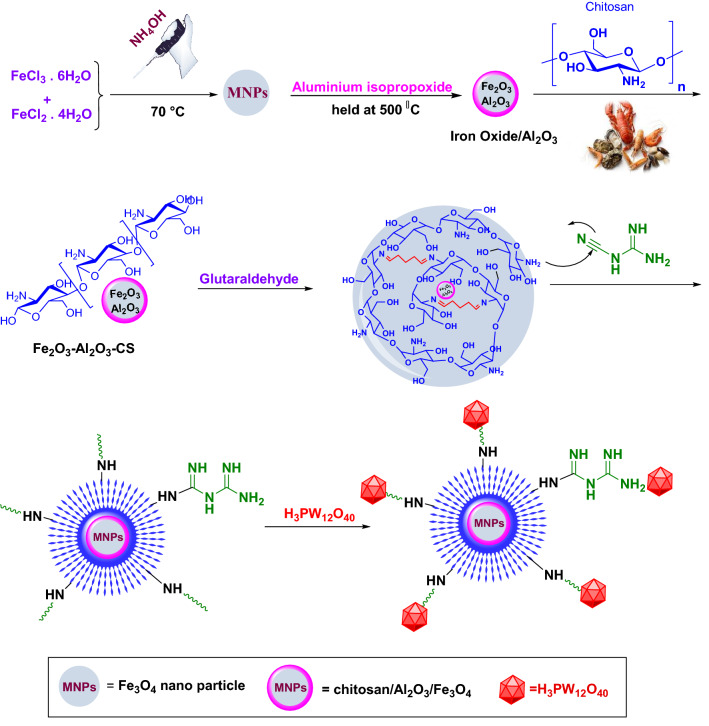
The representation of the fabrication of HPA@Fe_3_O_4_–Al_2_O_3_/CS–CG.

For the synthesis of HPW embedded *Fe*_*3*_*O*_*4*_–*Al*_*2*_*O*_*3*_–*CS*–*CG* composite, 1 g of as-prepared Fe_3_O_4_–Al_2_O_3_–CS–CG was suspended in a solution of distilled water and ethanol (1:2) and stirred for 10 min. Afterward, a solution of H_3_PW_12_O_40_ (5 wt%) was added dropwise to the suspension. The resultant mixture was then stirred at 60 °C for 2 h. Finally, the precipitate was separated by a magnet, washed several times with water, and dried in the oven at 70 °C. The possible preparation mechanism is illustrated in Fig. 2.

### Catalytic reactions

#### General procedure for the synthesis of 6-amino-8-aryl-7-cyano-8H-[1,3]dioxolo-[4,5-g]-chromenes

**Figure 3:**
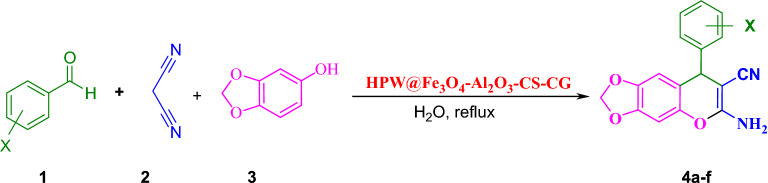
Synthesis of 6-amino-8-aryl-7-cyano-8*H*-[1, 3] dioxolo-[4,5-g]-chromene.

0.04 g of HPW@Fe_3_O_4_–Al_2_O_3_–CS–CG was added to a solution containing an appropriate aromatic aldehyde (1 mmol), malononitrile (1 mmol), and 3,4-methylene-dioxy-phenol (1 mmol) in H_2_O (5 mL), and stirred under reflux for a specific time (10–30 min). The reaction was monitored by TLC (Petroleum ether:ethyl acetate = 8:2). After the completion of the reaction, the mixture was cooled to room temperature and the catalyst was easily separated by an external magnet. The catalyst was reused several times without any special treatment and with no loss of appreciable activity at least in four successive runs. The precipitated solid was filtered off and washed with water to obtain the pure products, where any other purification process was not required (Fig. 3).

#### Synthesis of 4H-benzo [b]pyrans

**Figure 4:**
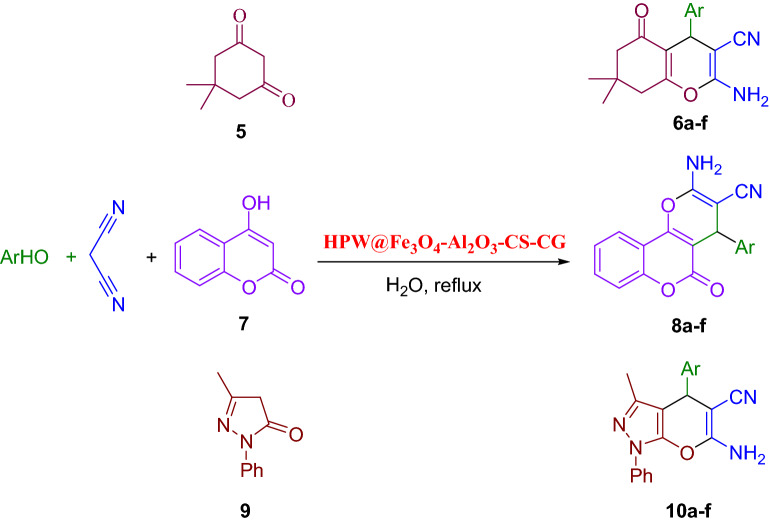
Synthesis of 2-amino-4*H*-chromene derivatives using HPW@Fe_3_O_4_–Al_2_O_3_–CS–CG.

0.04 g of HPW@Fe_3_O_4_–Al_2_O_3_–CS–CG was added to a solution of an aromatic aldehyde, malononitrile, and dimedone/4-Hydroxy coumarine/3-methyl-1-phenyl-pyrazole-5-one (1 mmol of each compound) in H_2_O (5 mL) and stirred for a specific time under heating conditions. After completion of the reaction, which was monitored by TLC, the mixture was cooled to room temperature and the catalyst was separated subsequently. The solid product was collected by filtration, washed with the solution of water and ethanol, and purified by recrystallization from ethanol (Fig. 4).

## Results and discussion

### Characterization of synthesized nanocatalyst

**Figure 5:**
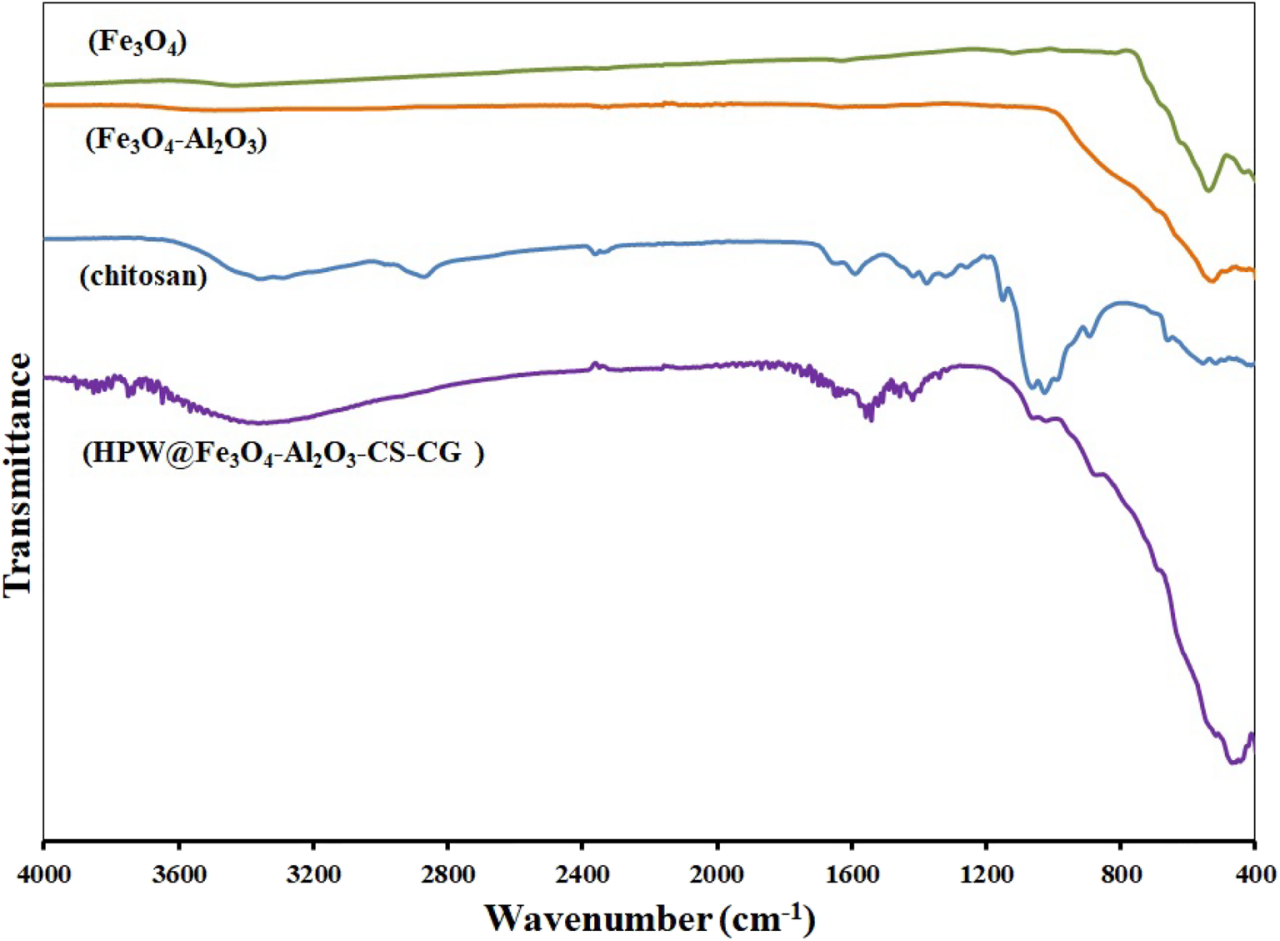
The FTIR spectrum of (**a**) Fe_3_O_4_, (**b**) Al_2_O_3_, (**c**) chitosan and (**d**) HPW@Fe_3_O_4_–Al_2_O_3_–CS–CG.

The designed HPW@Fe_3_O_4_–Al_2_O_3_–CS–CG catalyst was identified by Fourier Transform Infrared (FTIR) methods and the obtained spectrum is shown in Fig. 5. As can be seen, the peak at 518 cm^−1^ and wide peak at 674 cm^−1^ are related to Fe–O, Al–O, and Fe–Al–O showing the cross-linking of chitosan and iron oxide/Al_2_O_3_ core–shell spheres^16,51^. The peak of C=N at 1630 cm^−1^ was attributed to the presence of cyanoguanidine and glutaraldehyde as cross-linkers^52^. The broad band at 3150–3700 cm^−1^ and the peaks at 1651, 1557 cm^−1^, and 1387 cm^−1^ are attributed to hydroxyl (O–H) stretching overlapped with N–H stretch, amide I, II, and CH_3_ symmetrical angular deformation respectively^53^. The HPW structure consists of tungsten atoms, linked by oxygen atoms, while the phosphorus atoms are at the center of the tetrahedron. The three characteristic absorption bands of HPW are appeared at 1060 cm^−1^ (P–O in the central PO_4_ tetrahedron), 1012 cm^−1^ (W=O in the exterior), and 879 cm^−1^ (W–O_*b*_–W bridges between corner sharing octahedron)^54^.

**Figure 6:**
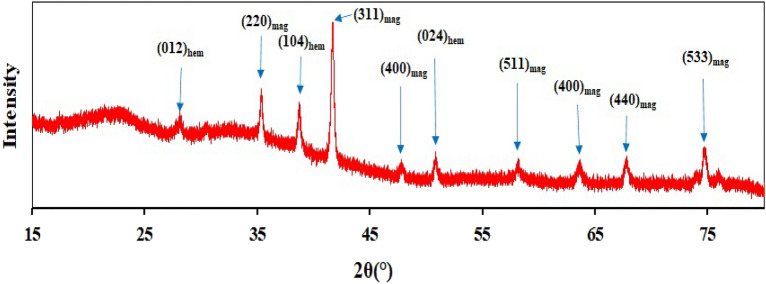
XRD spectrum of the prepared HPW@Fe_3_O_4_–Al_2_O_3_–CS–CG catalyst.

The crystallinity, phase components, and molecule structure of the HPW@Fe_3_O_4_–Al_2_O_3_–CS–CG catalyst were analyzed by XRD, as shown in Fig. 6. It shows the diffraction peaks of Fe_3_O_4_ nanoparticles at 30.3°, 35.7°, 43.5°, 53.8°, 57.5°, 63.3°, 67°, and 74.1° with a typical face-centered cubic structure^15,16^. The characteristic diffraction peaks of cubic face-centered magnetite (Fe_3_O_4_) and rhombohedral hematite (Fe_2_O_3_), as shown in the Figure, indicate the presence of magnetite nanoparticles in the prepared catalyst. On the other hand, the amorphous hump in the 2θ range of 15 to 25° is corresponded to the amorphous chitosan/HPW phase.

**Figure 7:**
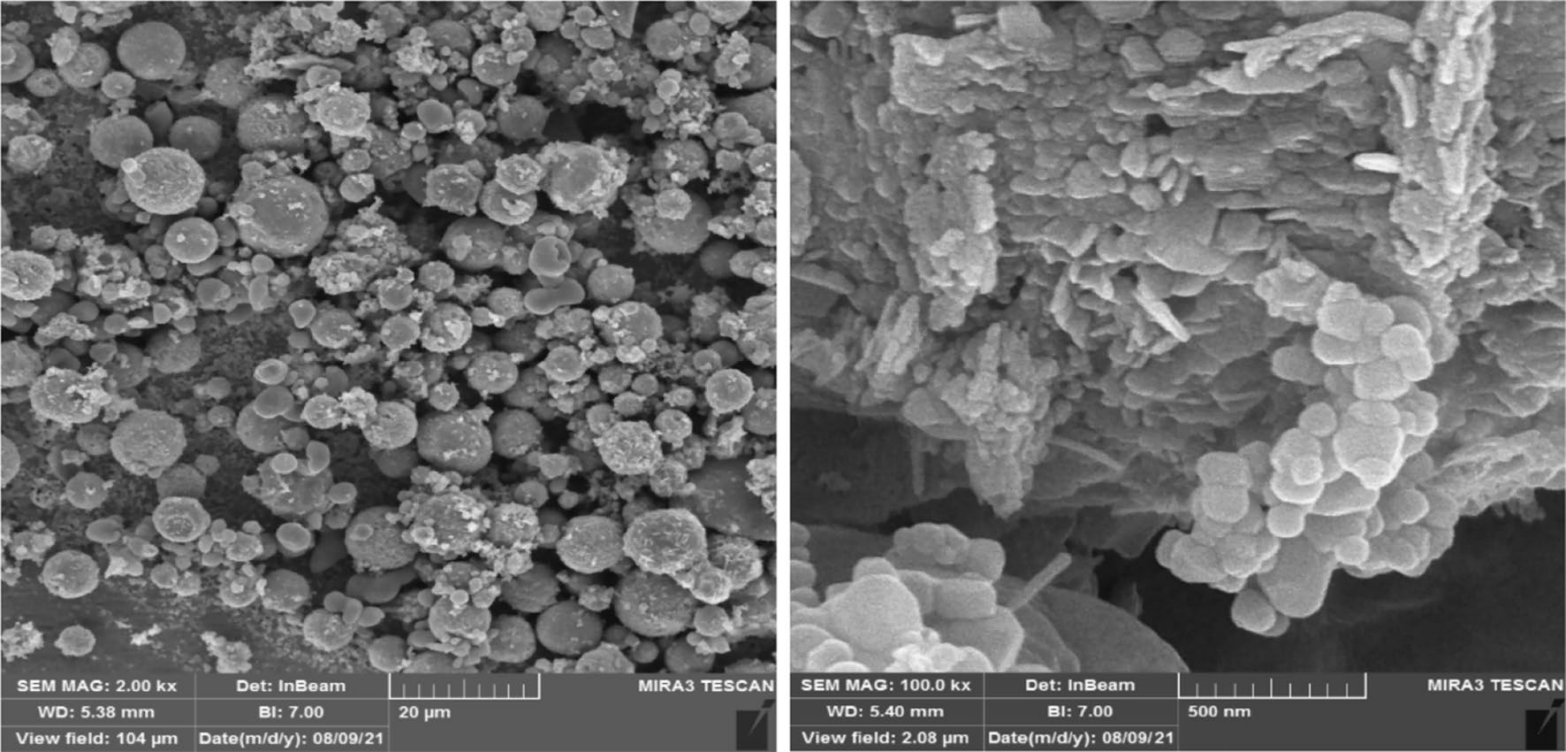
The SEM images of prepared HPW@Fe_3_O_4_–Al_2_O_3_–CS–CG catalyst.

Moreover, the SEM images of the HPW@Fe_3_O_4_–Al_2_O_3_–CS–CG catalyst are shown in Fig. 7 at different magnifications. Before observation, the catalyst was sputtered with thin gold layers to enhance its electrical conductivity. SEM images show that the final product exhibits the aggregation of particles in the range of 100–500 nm, which can be resulted from the embedding of magnetic iron oxide/Al_2_O_3_ particles inside the cross-linked chitosan.

**Figure 8:**
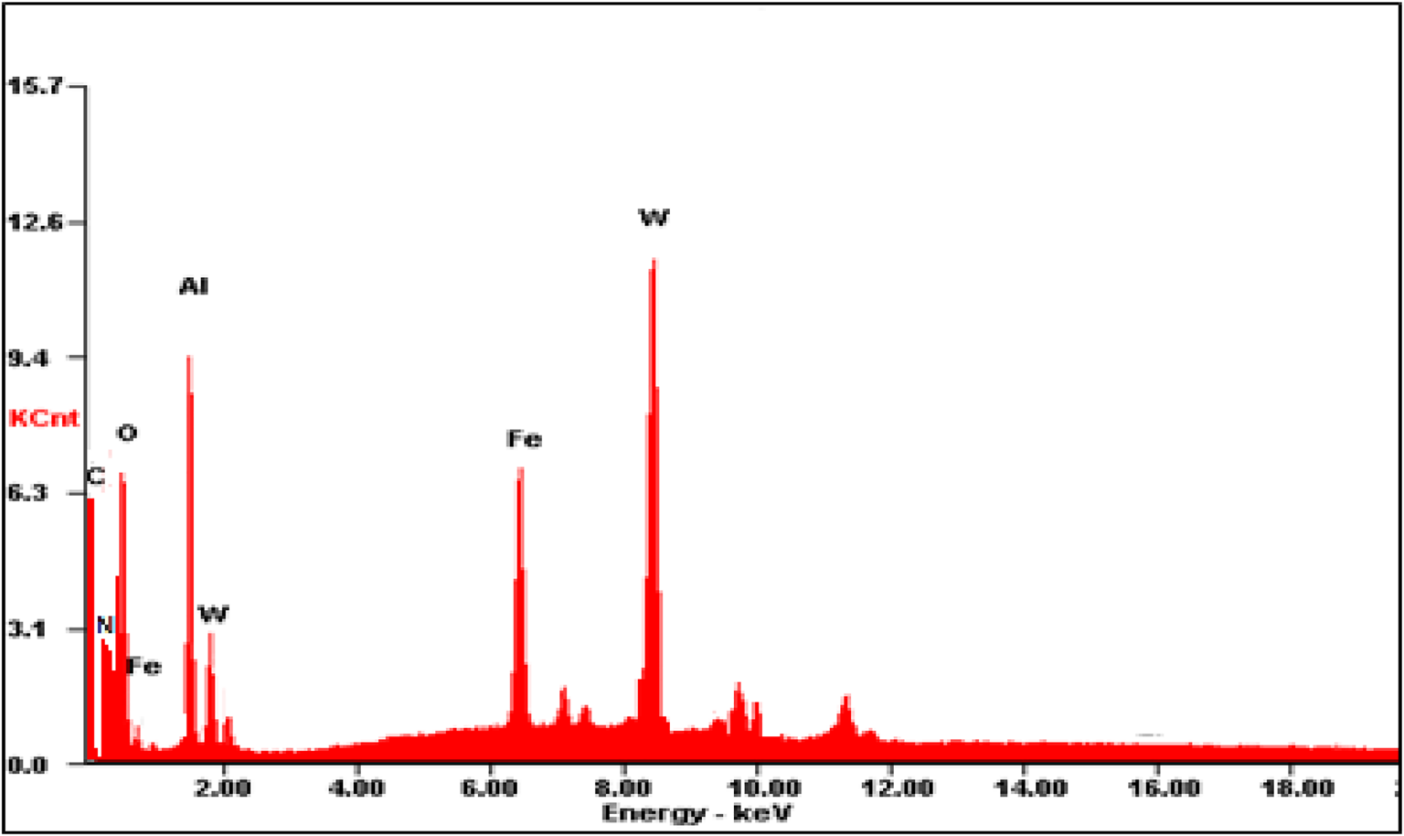
EDS analysis of HPW@Fe_3_O_4_–Al_2_O_3_–CS–CG catalyst.

Furthermore, the EDS analysis was also applied to examine the elemental analysis of HPW@Fe_3_O_4_–Al_2_O_3_–CS–CG catalyst. The spectrum is shown in Fig. 8. The results confirmed the well dispersion of phosphotungstic acid in the catalyst structure.

**Figure 9:**
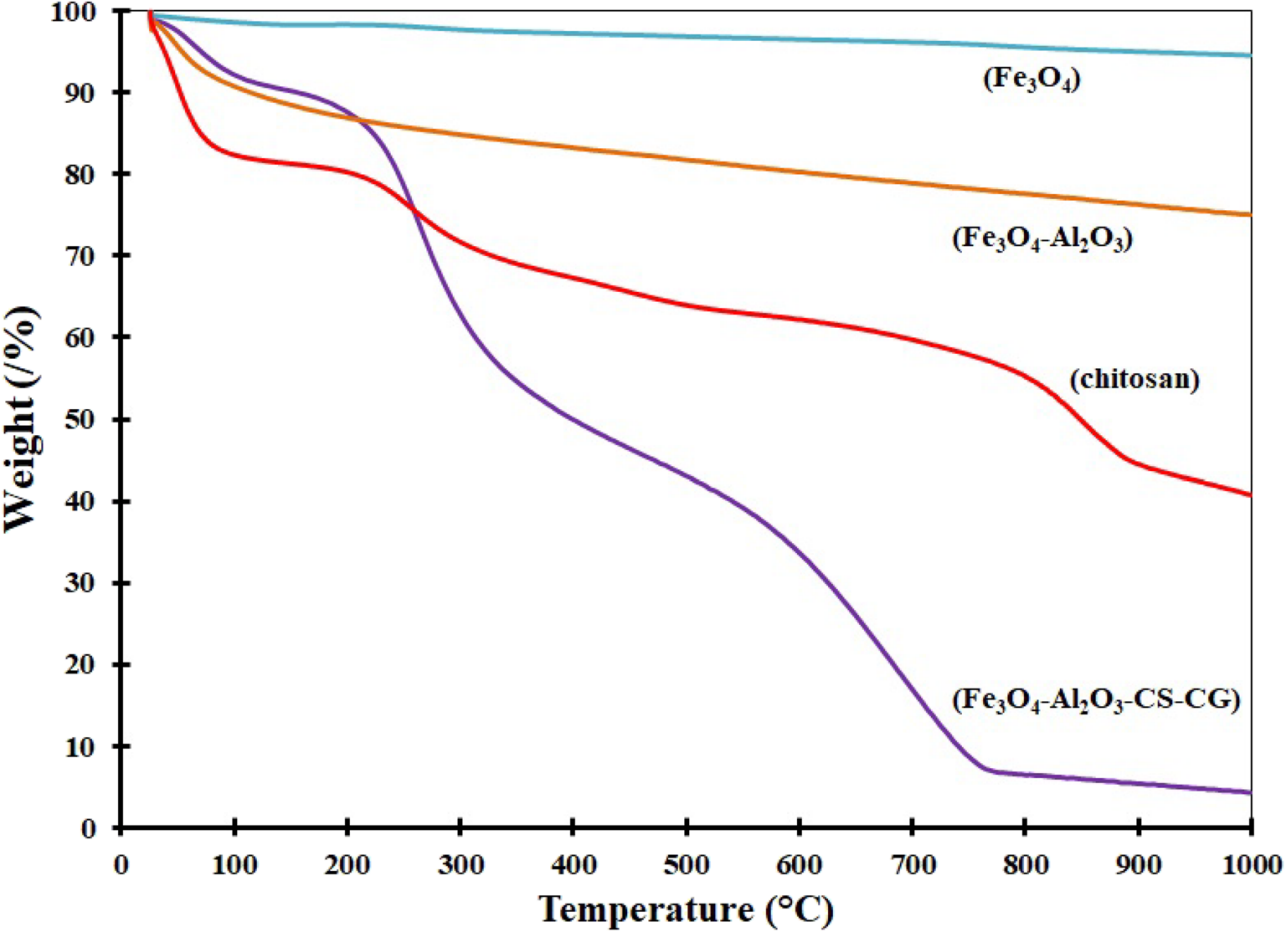
Thermogravimetry curves of Fe_3_O_4_, Al_2_O_3_, chitosan, and HPW@Fe_3_O_4_–Al_2_O_3_–CS–CG.

The TGA curves of the Fe_3_O_4_, Al_2_O_3_, chitosan, and HPW@Fe_3_O_4_–Al_2_O_3_–CS–CG are shown in Fig. 9. The thermogravimetric curve of Fe_3_O_4_ nanoparticles shows a weight loss of ~ 6.5%. The first weight loss up to the temperature of 150° is attributed to the removal of water from the surface of the nanoparticles, and the rest is related to the removal of water from the structure. The TGA curve of Al_2_O_3_ nanoparticles showed two weight loss stages related to the loss of moisture from the surface of the Al_2_O_3_ layer (150 °C) and exit of the structured water (200–1000 °C). On the other hand, the water removal occurs in the range of 100 °C in chitosan, followed by the deacetylation process that causes weight loss between 200 and 470 °C. In the case of HPW@Fe_3_O_4_–Al_2_O_3_–CS–CG, apart from weight loss due to water removal, the second stage of degradation was observed at 260 °C with a mass loss of ~ 40%. The temperature of the second weight loss of catalyst is lower than that of chitosan, showing that the new compound is more thermally stable. Finally, the organic polymer is almost wholly decomposed at 750 °C^16^.

### Effects of the catalyst structure

To establish the possibility of our strategy for the synthesis of 6-amino-8-aryl-7-cyano-8*H*-[1,3]dioxolo-[4,5-g]-chromene, as well as optimizing the reaction conditions, the condensation of 3,4-methylene-dioxy-phenol, benzaldehde, and malononitrile were studied in the presence of a different solvent, temperature, and also the amount of catalyst. The results are summarized in Table 1. As can be seen, the desired product was produced with a negligible yield in the absence of HPW@Fe_3_O_4_-Al_2_O_3_-CS-CG catalyst (Table 1, Entry 1). After that, the effect of several solvents and temperature were explored. As listed in Table 1, the results demonstrated that the presence of catalyst and solvent were significantly essential to accomplish the reaction. After optimizing the catalyst loading and temperature, the effect of some solvents was assessed. The optimal reaction condition was obtained using 0.04 g of HPW@Fe_3_O_4_-Al_2_O_3_-CS-CG catalyst under reflux in water (Table 1, Entry 11).

**Table 1:** The results of 6-Amino-7-cyano-8-phenyl-8H-[1,3]dioxolo [4,5-g]-chromene synthesis under heating conditions.

Entry	Reaction condition	Catalyst/(g)	Time (min)	Yield (%)
1	H_2_O/reflux	–	60	10
2	H_2_O/reflux	H_3_PW_12_O_40/_0.04	30	88
3	H_2_O/reflux	Fe_3_O_4 /_0.04	30	89
4	EtOH, r.t	HPW@Fe_3_O_4_–Al_2_O_3_–CS–CG/0.04	30	80
5	EtOH, reflux	HPW@Fe_3_O_4_–Al_2_O_3_–CS–CG/0.04	20	90
6	CH_3_CN, reflux	HPW@Fe_3_O_4_–Al_2_O_3_–CS–CG/0.04	25	87
7	CHCl_3_, reflux	HPW@Fe_3_O_4_–Al_2_O_3_–CS–CG/0.04	30	80
8	Toluene, 80 °C	HPW@Fe_3_O_4_–Al_2_O_3_–CS–CG/0.04	30	87
9	H_2_O/r.t	HPW@Fe_3_O_4_–Al_2_O_3_–CS–CG/0.04	30	80
10	H_2_O/50 °C	HPW@Fe_3_O_4_–Al_2_O_3_–CS–CG/0.04	20	94
11	H_2_O/reflux	HPW@Fe_3_O_4_–Al_2_O_3_–CS–CG/0.04	15	97
12	H_2_O/reflux	HPW@Fe_3_O_4_–Al_2_O_3_–CS–CG/0.06	15	97
13	H_2_O/reflux	HPW@Fe_3_O_4_–Al_2_O_3_–CS–CG/0.02	20	95

Reaction conditions: HPW@Fe_3_O_4_–Al_2_O_3_–CS–CG, Solvent (5 ml).

So, the efficiency of this approach was studied in a wide variety of substituted pyranochromene derivatives synthesis under the obtained optimized conditions. Tables 2 and 3 show the summarized results. They reveal the excellent product yields of all reactions and accommodated a wide range of aromatic aldehydes bearing both electron-withdrawing and electron-donating substituents. The pure product in all cases was isolated by simple filtration and washing with water without any purification process.

**Table 2:** 6-Amino-8-aryl-7-cyano-8H-[1,3]dioxolo-[4,5-g] chromenes synthesis in the presence of HPW@Fe_3_O_4_–Al_2_O_3_–CS–CG.

Entry	Ar	Product	Time (min)	Yield (%)^a^	M. P. (Lit. mp)^55^ (°C)
1	C_6_H_5_	4a	15	97	208–210 (207)
2	3-NO_2_C_6_H_4_	4b	20	96	213–216 (212)
3	4-CH_3_C_6_H_4_	4c	20	95	207–210 (208)
4	4-CH_3_OC_6_H_4_	4d	25	96	195–197 (197)
5	4-ClC_6_H_4_	4e	12	97	203–205 (205)
6	3-BrC_6_H_4_	4f.	15	96	227–229 (227)

**Table 3:** Synthesis of 2-amino-4*H*-chromene derivatives in the presence of HPW@Fe_3_O_4_–Al_2_O_3_–CS–CG.

Entry	Ar	Product	Time (min)	Yield (%)^a^	M. P. (Lit. mp) (°C)^39,56^
1	C_6_H_5_	6a	15	98	228–230 (227)
2	3-NO_2_C_6_H_4_	6b	12	94	207–209 (209)
3	4-CH_3_C_6_H_4_	6c	15	94	212–213 (212)
4	4-CH_3_OC_6_H_4_	6d	18	95	201–202 (202)
5	4-ClC_6_H_4_	6e	8	97	206–208 (207)
6	4-BrC_6_H_4_	6f.	10	95	199–200 (197)
7	C_6_H_5_	8a	20	95	260–262 (261)
8	3-NO_2_C_6_H_4_	8b	25	94	258–259 (258)
9	4-NO_2_C_6_H_4_	8c	25	95	259–260 (260)
10	2,4-Cl_2_C_6_H_3_	8d	35	92	254–257 (255)
11	4-ClC_6_H_4_	8e	20	96	266–268 (265)
12	2,3-Cl_2_C_6_H_3_	8f.	40	91	280–283 (280)
13	C_6_H_5_	10a	30	97	166–170 (167)
14	3-NO_2_C_6_H_4_	10b	35	94	189–190 (190)
15	4-NO_2_C_6_H_4_	10c	30	96	194–196 (195)
16	4-CH_3_OC_6_H_4_	10d	32	93	174–175 (174)
17	4-ClC_6_H_4_	10e	27	95	175–177 (174)
18	4-MeC_6_H_4_	10f.	35	90	177–179 (178)

### Recycling of the catalyst

**Figure 10:**
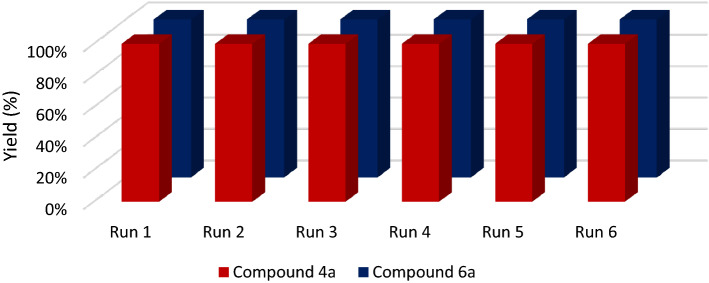
Reusability of the catalysts in the synthesis of compounds 4a and 6a.

As one of the most important applicability features of catalyst, the reusability of the HPW@Fe_3_O_4_–Al_2_O_3_–CS–CG catalyst was also studied. In this regard, the catalyst was separated by an external magnet after completing the model reaction and washed several times with acetone. It was recycled to the reaction catalyst for the second or even more reaction runs, and the yields are presented in Tables 2 and 3. The results show that the catalytic performance of HPW@Fe_3_O_4_–Al_2_O_3_–CS–CG was almost the same as those of fresh catalyst, after 6 runs of reaction (see Fig. 10).

### Uniqueness of our protocol

To demonstrate the exclusivity of HPW@Fe_3_O_4_–Al_2_O_3_–CS–CG, as a heterogeneous catalyst in the synthesis of [1, 3]dioxolo-[4,5-g]-chromene derivatives, the obtained results in the optimized model reaction conditions were compared with the reported ones in the literature, as displayed in Table 4. Evidently, the HPW@Fe_3_O_4_–Al_2_O_3_–CS–CG is the most efficient catalyst among them in terms of reaction time and yield. Significantly, the reported synthetic paths have some limitations, such as requiring extreme temperature or long duration, large amounts of the catalyst, and most importantly, using hazardous solvents.

**Table 4:** Comparison of the catalytic activity of HPW@Fe_3_O_4_–Al_2_O_3_–CS–CG catalyst with reported results in the literature.

Entry	Reaction condition	Time (h)	Yield (%)	References
1	Piperidine/ethanol	67	81	^57^
2	Aluminum oxide/H_2_O	0.15	93	^55^
3	TiO_2_ NPs/H_2_O	6	94	^58^
4	Cetyltrimethylammonium chloride/H_2_O	6	92	^59^
5	Triethylamine/ethanol	40	65	^60^
6	HPW@Fe_3_O_4_–Al_2_O_3_**–**CS–CG	0.12	97	This work

### Product characterization data^39,56^

6-Amino-7-cyano-8-Phenyl-8H-[1,3]dioxolo-[4,5-g] chromene (4a):

M.p. 207–210 °C IR (KBr) υ_max_ = 3441, 3349, 3073, 2910, 2184,1656,1630, 1595, 1479, 1352, 1250, 1089, 1035, 811, 763 cm^−1^. ^1^H-NMR (DMSO-d_6_, 500 MHz) δ_H_ = 4.66 (s, 1H, H-8), 5.34 (s, 1H, CH-2), 5.93 (s, 1H, CH-2'), 5.96 (s, 1H, Ar), 6.39(s, 1H, CH), 6.56 (s, 1H, CH), 6.81 (s, 2H, NH_2_), 6.87–6.88 (d, 2H, J = 8.56), 7.09–7.11 (d, 2H, J = 8.56) ppm.

6-Amino-7-cyano-8-(4-Methoxyphenyl)-8H-[1, 3] dioxolo-[4,5-g] chromene (4b):

M.p. 197–201 °C IR (KBr) υ_max_ = 3440, 3341, 3204, 2972, 2180, 1663, 1606, 1507, 1405, 1247, 1179, 1092, 804, 769 cm^−1^. ^1^H-NMR (DMSO d_6_, 500 MHz) δ_H_ = 3.72 (s, 3H, CH_3_), 4.57 (s, 1H, H-8), 5.94 (s, 1H, CH), 6.0007 (s, 1H, CH), 6.50 (s, 1H, CH), 6.66 (s, 1H, CH), 6.81 (s, 2H, NH_2_), 6.88–6.87 (d, 2H, J = 8.56), 7.09–7.11 (d, 2H, J = 8.56) ppm. ^13^C-NMR (DMSO-d_6_, 125 MHz) δ = 41.011, 55.92, 56.88, 98.52, 102.47, 108.20, 114.88, 116.76, 121.44, 129.20, 138.96, 143.49, 144.85, 147.50, 158.97, 161.08 ppm.

6-Amino-7-cyano-8-(3-Nitrophenyl)-8H-[1, 3] dioxolo-[4,5-g] chromene (4c):

M.p. 212–215 °C IR (KBr) υ_max_ = 3440, 3348, 3203, 2906, 2183, 1657, 1630, 1595, 1402, 1250, 1180, 1088, 806, 763 cm^−1^. ^1^H-NMR (DMSO d_6_, 500 MHz) δ_H_ = 4.91 (s, 3H, CH_3_), 5.96 (s, 1H, H-8), 6.021–6.022 (d, 1H, j = 0.72), 6.61 (s, 1H, CH), 6.72(s, 1H, CH), 7.03 (s, 2H, NH_2_), 7.63–7.69 (m, 2H), 8.05–8.06(m, 1H, Ar), 8.1–8.12 (m, 1H, Ar)ppm. ^13^C-NMR (DMSO-d_6_, 125 MHz) δ = 41.005, 55.694, 98.792, 102.667, 108.135, 115.205, 121.095, 122.576, 122.885, 131.293, 135.046, 143.704, 145.145, 148.018, 148.868, 149.0, 161.501 ppm.

6-Amino-7-cyano-8-(4-Methylphenyl)-8H-[1, 3] dioxolo-[4,5-g] chromene (4d):

M.p. 208–211 °C IR (KBr) υ_max_ = 3451, 3335, 3220, 2887, 2193, 1670, 1602, 1502, 1412, 1243, 1184, 1091, 844, 783 cm^−1^. ^1^H-NMR (DMSO d_6_, 500 MHz) δ_H_ = 2.26 (s, 3H, CH3), 4.57 (s, 1H, H-8), 5.94 (s, 1H, CH), 6.004 (s, 1H, CH), 6.51 (s, 1H, CH), 6.66 (s, 1H, CH), 6.83 (s, 2H, NH_2_), 7.06–7.08 (d, 2H, J = 7.65), 7.11–7.13(d, 2H, J = 7.89) ppm. ^13^C-NMR (DMSO-d_6_, 125 MHz) δ = 21.45, 41.20, 56.69, 98.54, 102.48, 108.19, 116.59, 121.41, 128.05, 130.05, 136.77, 143.53, 143.92, 144.85, 147.54, 161.17 ppm.

6-Amino-7-cyano-8-(4-Chlorophenyl)-8H-[1,3]dioxolo-[4,5-g] chromene (4e):

M.p. 205–208 °C IR (KBr) υ_max_ = 3449, 3338, 3256, 2887, 2191, 1668, 1600, 1482, 1411, 1244, 1183, 1093, 838, 791 cm^−1^. ^1^H-NMR (DMSO d_6_, 500 MHz) δ_H_ = 4.67 (s, 1H, H-8), 5.95 (s, 1H, CH), 6.01 (s, 1H, CH), 6.54 (s, 1H, CH), 6.68(s, 1H, CH), 6.92 (s, 2H, NH_2_), 7.21–7.22 (d, 2H, J = 8.41), 7.37–7.39(d, 2H, J = 8.41) ppm. ^13^C-NMR (DMSO-d_6_, 125 MHz) δ = 56.17, 98.65, 102.57, 108.126, 115.85, 121.25, 129.51, 130.04, 132.29, 143.6, 144.99, 145.79, 147.77, 161.26 ppm.

6-Amino-7-cyano-8-(3-Bromophenyl)-8H-[1, 3]-dioxolo-[4,5-g] chromene (4f.):

M.p. 227–230 °C IR (KBr) υ_max_ = 3449, 3325, 3204, 2894, 2191, 1658, 1595, 1478, 1431, 1249, 1177, 1084, 839, 767 cm^−1^. ^1^H-NMR (DMSO d_6_, 500 MHz) δ_H_ = 4.68 (s, 1H, H-8), 5.967–5.969 (d, 1H, CH), 6.021–6.022 (d, 1H, CH), 6.59 (s, 1H, CH), 6.70(s, 1H, CH), 6.96 (s, 2H, NH_2_), 7.19–7.21 (d, 1H, J = 7.76), 7.28–7.31 (t, 1H, J = 8.41), 7.375–7.379 (d, 1H, J = 1.69), 7.42–7.43 (t, 1H, J = 0.96), 7.44–7.446 (m, 1H) ppm. ^13^C-NMR (DMSO-d_6_, 125 MHz) δ = 41.21, 55.97, 98.70, 102.61, 108.09, 115.65, 121.21, 122.78, 127.33, 130.64, 130.73, 131.86, 143.61, 145.04, 147.86, 149.56, 161.39 ppm.

#### 2-Amino-4-(3-nitrophenyl)-3-cyano-4H,5H-pyrano[3,2-c]chromene-5-one (8b)

IR (KBr) υ_max_ = 3404, 3322,3194, 2202, 1703, 1672, 1531, 1349 cm^−1^,^1^HNMR (DMSO-d_6_,500 MHz): δ_H_ = 4.74 (1H, s, H_4_), 7.44 (1H, d, J = 6.7 Hz), 7.51 (1H, t, J = 7.6 Hz), 7.56 (2H, brs, NH_2_), 7.64 (1H, t, J = 7.6 Hz), 7.73 (1H, d, J = 7.5, 1.3 Hz), 7.82 (1H, d, J = 6.8 Hz, H2’), 7.92 (1H, dd, J = 6.8), 8.12 (1H, dd, J = 8.4), 8.14 (1H, s, H6’) ppm.

#### 2-Amino-4-(4-nitrophenyl)-3-cyano-4H,5H-pyrano[3,2-c]chromene-5-one (8c)

IR (KBr):) υ_max_ = 3482,3432, 3371, 3335, 2195, 1718, 1673, 1607, 1506, 1374,1306 cm^−1^ ^1^HNMR (DMSO-d_6_,500 MHz): δ_H_ = 4.68 (1H, s, H-4), 7.47 (1H,d, J = 8.3 Hz), 7.52 (1H, t, J = 7.7 Hz), 7.57 (2H, bs, NH_2_), 7.60 (2H, d, J = 8.0 Hz), 7.74 (1H, t, J = 7.8 Hz), 7.91 (1H,d, J = 7.8 Hz), 8.18 (2H, d, J = 8.3 Hz) ppm.

#### 2-Amino-4-(2,4-dichlorophenyl)-3-cyano-4H,5H-pyrano[3,2-c]chromene-5-one (8d)

IR (KBr):) υ_max_ = 3463, 3295,3163, 3070, 2198, 1715, 1674, 1590 cm^−1^; ^1^HNMR (DMSO-d_6_,500 MHz): δ_H_ = 4.99 (1H, s, H-4), 7.36 (1H, dd,J = 8.3),7.40 (1H, d, J = 8.3 Hz), 7.41 (2H, brs, NH_2_), 7.46 (1H, d,J = 8.3 Hz), 7.51 (1H, t, J = 7.7 Hz), 7.56 (1H, d, J = 2.1 Hz), 7.73 (1H, t, J = 8.2 Hz), 7.92 (1H, d, J = 8.9 Hz) ppm.

#### 2-Amino-4-(2,3-dichlorophenyl)-3-cyano-4H,5H-pyrano[3,2-c]chromene-5-one (8f.)

IR (KBr): ) υ_max_ = 3403, 3294,3179, 2198, 1710, 1672, 1601 cm^−1^; ^1^HNMR (DMSO-d_6_,500 MHz): δ_H_ = 5.09 (1H, s, H-4), 7.29–7.35 (2H, m, 7.47–7.55 (5H, m, H7,8,9 &NH_2_), 7.72–7.75 (1H, m, H6’), 7.92 (1H, dd, J = 7.8) ppm.

## Conclusion

In this study, a novel nanocatalyst was successfully prepared by heteropolyacid immobilization of cyanoguanidine modified magnetic chitosan composite and fully characterized by different analysis methods, including FTIR, XRD, TGA, SEM, and EDS. The catalytic activity of fabricated composite was examined in a one-pot three-component reaction, involving the diverse active methylene compounds, various aryl aldehydes, and malononitrile in water. The results revealed the efficient catalytic performance of composite, while all reactions proceeded smoothly and led to the formation of the corresponding pyranochromene derivatives in high to excellent yields.

## Supplementary Information

Supplementary Information.

## Data Availability

The raw/processed data that supports the findings of this study are available from the corresponding author upon reasonable request.
