# Supplementary material for: Heteropolyacid coupled with cyanoguanidine decorated magnetic chitosan as an efficient catalyst for the synthesis of pyranochromene derivatives
Source: Sci Rep. 2022 Oct 11;12:17027. doi: 10.1038/s41598-022-21196-2 (PMC9554034; doi:10.1038/s41598-022-21196-2)

IR Spectrum: 6-Amino-8-(3-nitrophenyl)-8H-[1,3]dioxolo[4,5-g]-chromene-7-carbonitrile, TABLE 3, Entry 2:

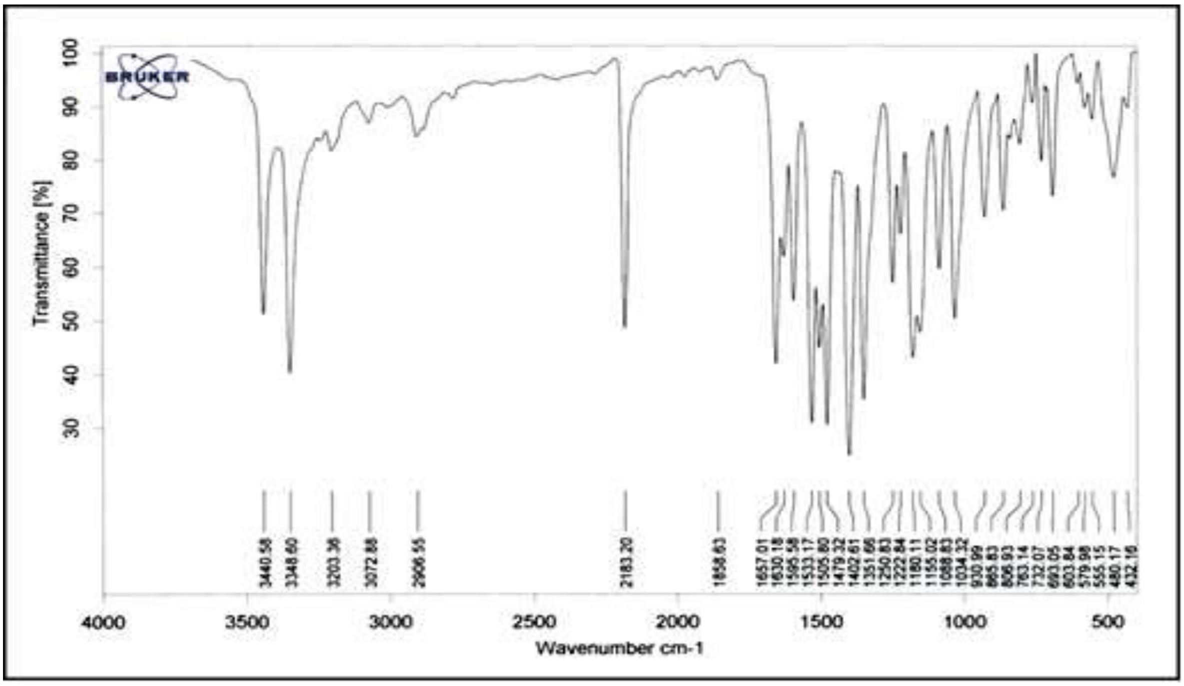

^{1}HNMR Spectrum: 6-Amino-8-(3-nitrophenyl)-8H-[1,3]dioxolo[4,5-g]-chromene-7-carbonitrile, TABLE 3, Entry 2:

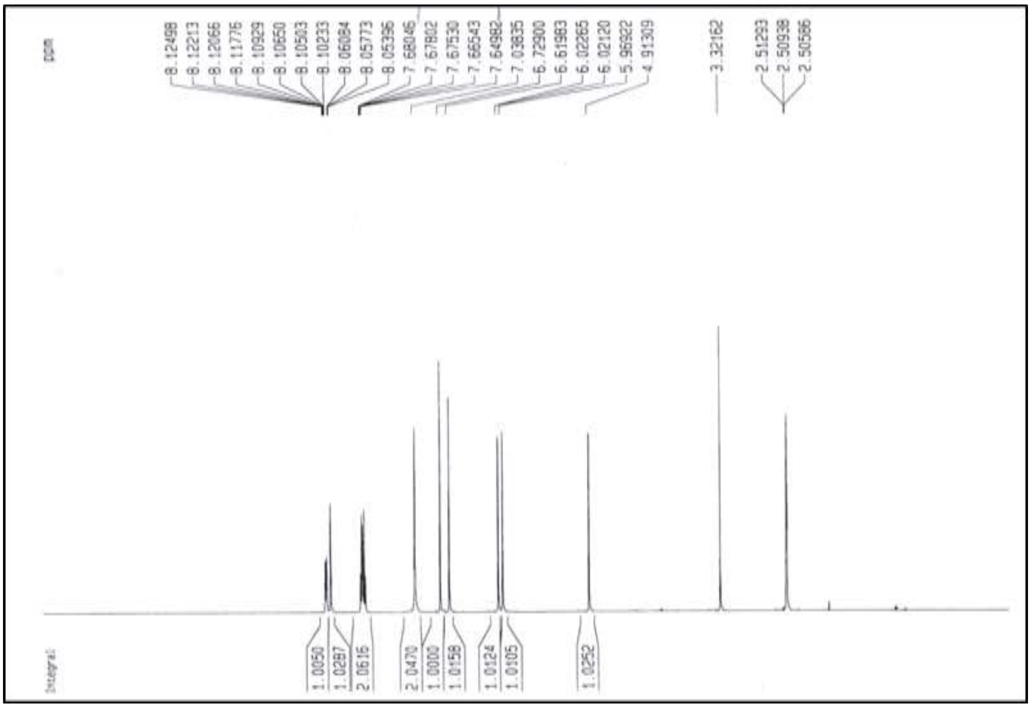

^{13}CNMR Spectrum: 6-Amino-8-(3-nitrophenyl)-8H-[1,3]dioxolo[4,5-g]-chromene-7-carbonitrile, TABLE 3, Entry 2:

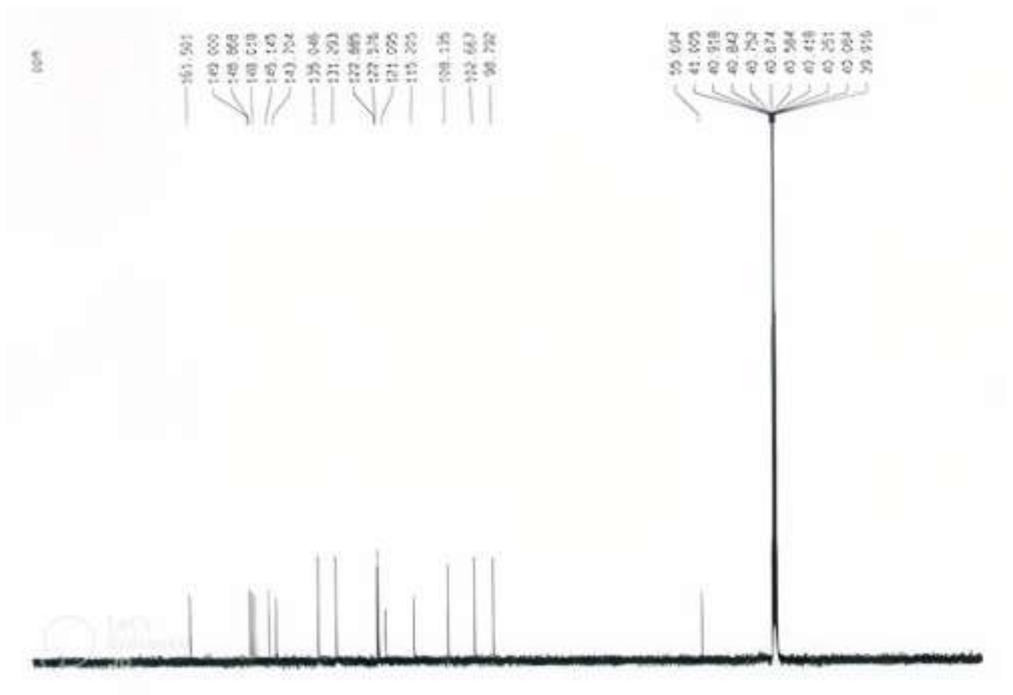

IR Spectrum: 6-Amino-8-(4-chlorophenyl)-8H-[1,3]dioxolo[4,5-g]-chromene-7-carbonitrile, TABLE 3, Entry 3:

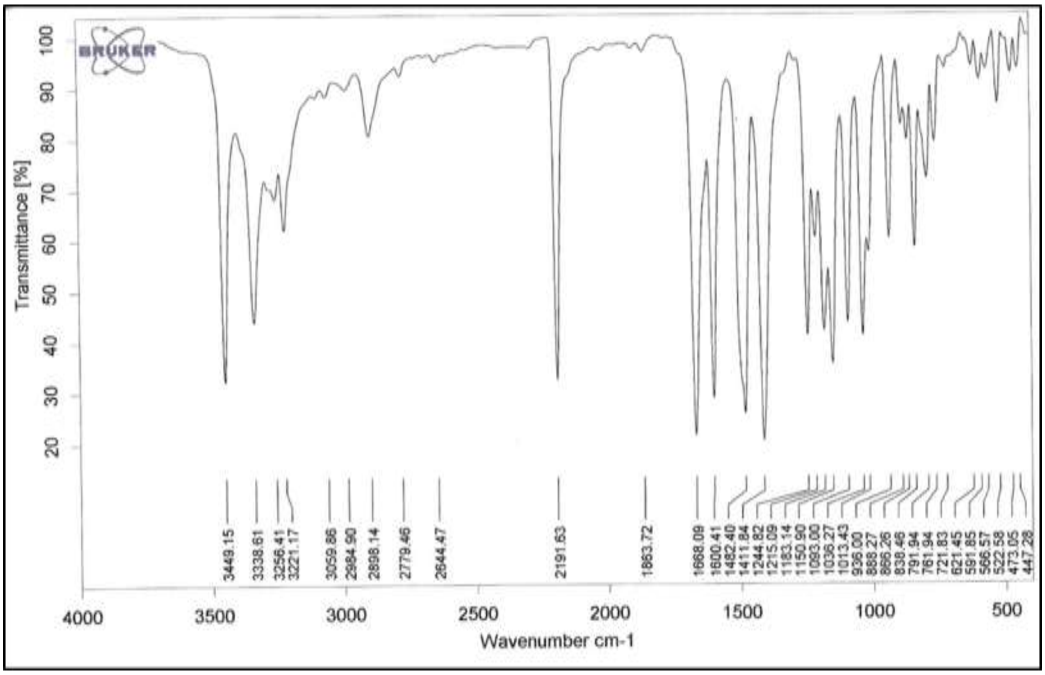

^{1}HNMR Spectrum: 6-Amino-8-(4-chlorophenyl)-8H-[1,3]dioxolo[4,5-g]-chromene-7-carbonitrile, TABLE 3, Entry 3:

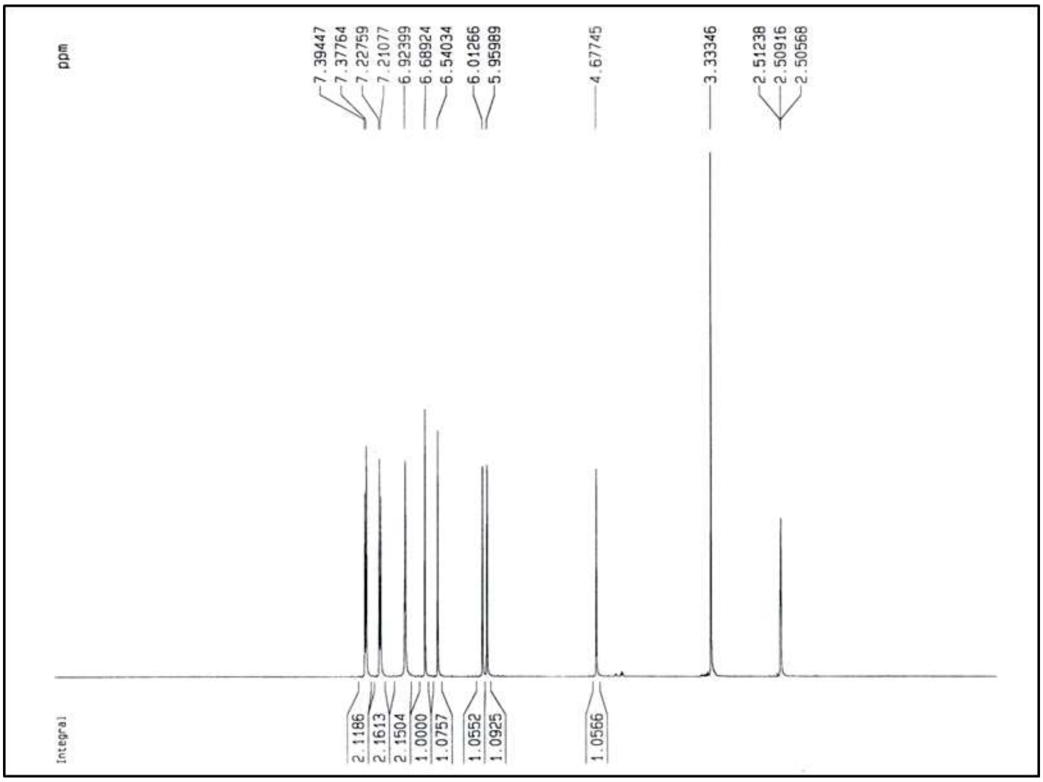

^{13}CNMR Spectrum: 6-Amino-8-(3-bromophenyl)-8H-[1,3]dioxolo[4,5-g]-chromene-7-carbonitrile, TABLE 3, Entry 3:

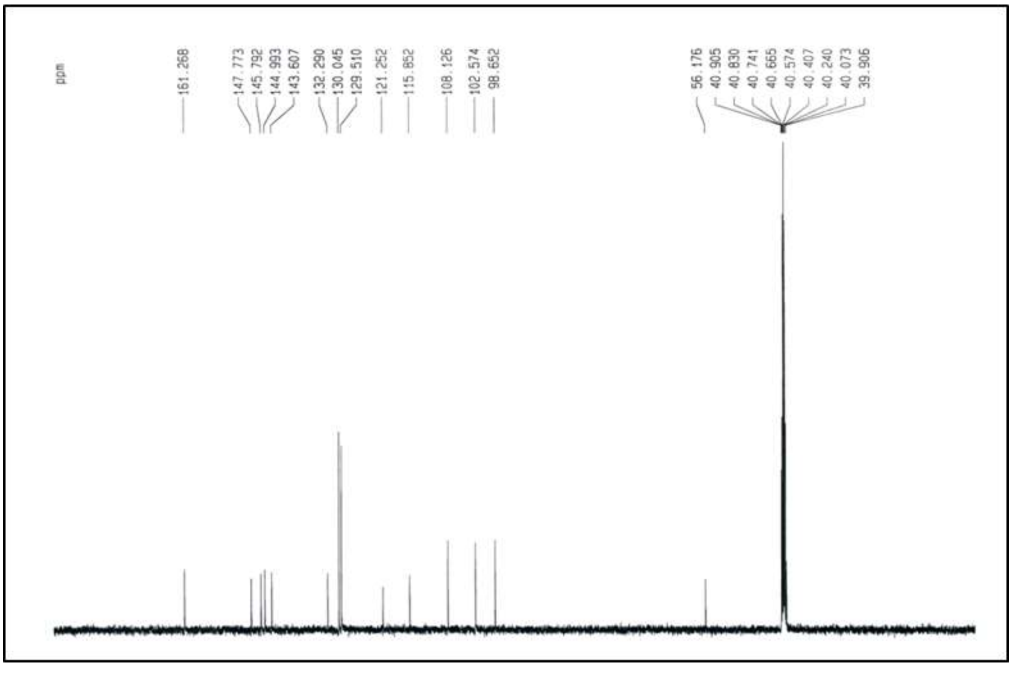

IR Spectrum: 6-Amino-8-(3-bromophenyl)-8H-[1,3]dioxolo[4,5-g]-chromene-7-carbonitrile, TABLE 3, Entry 6:

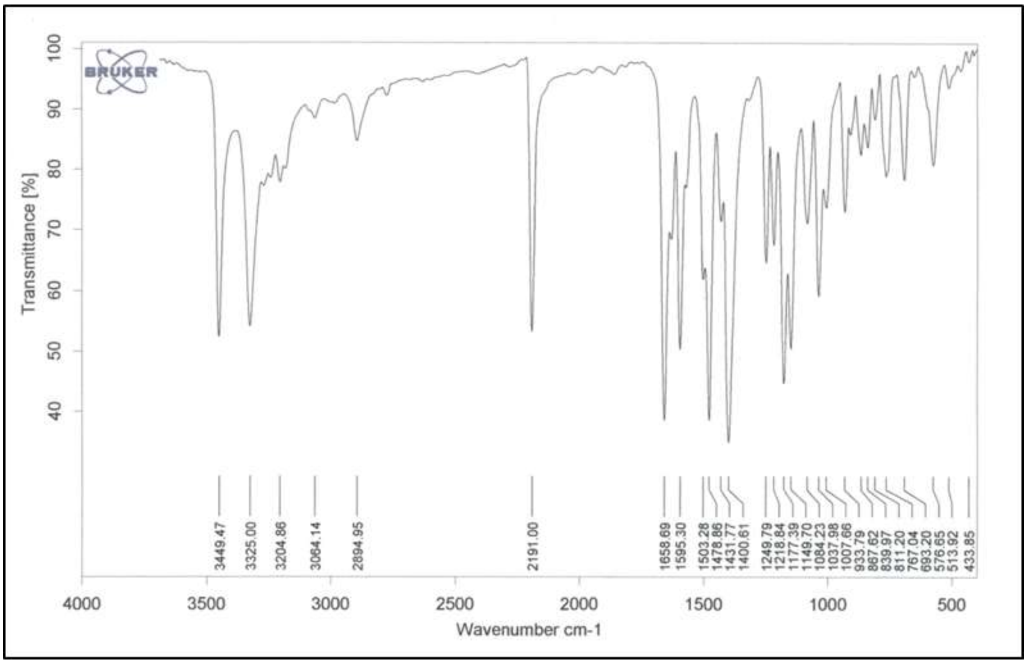

^{1}HNMR Spectrum: 6-Amino-8-(3-bromophenyl)-8H-[1,3]dioxolo[4,5-g]-chromene-7-carbonitrile, TABLE 3, Entry 6:

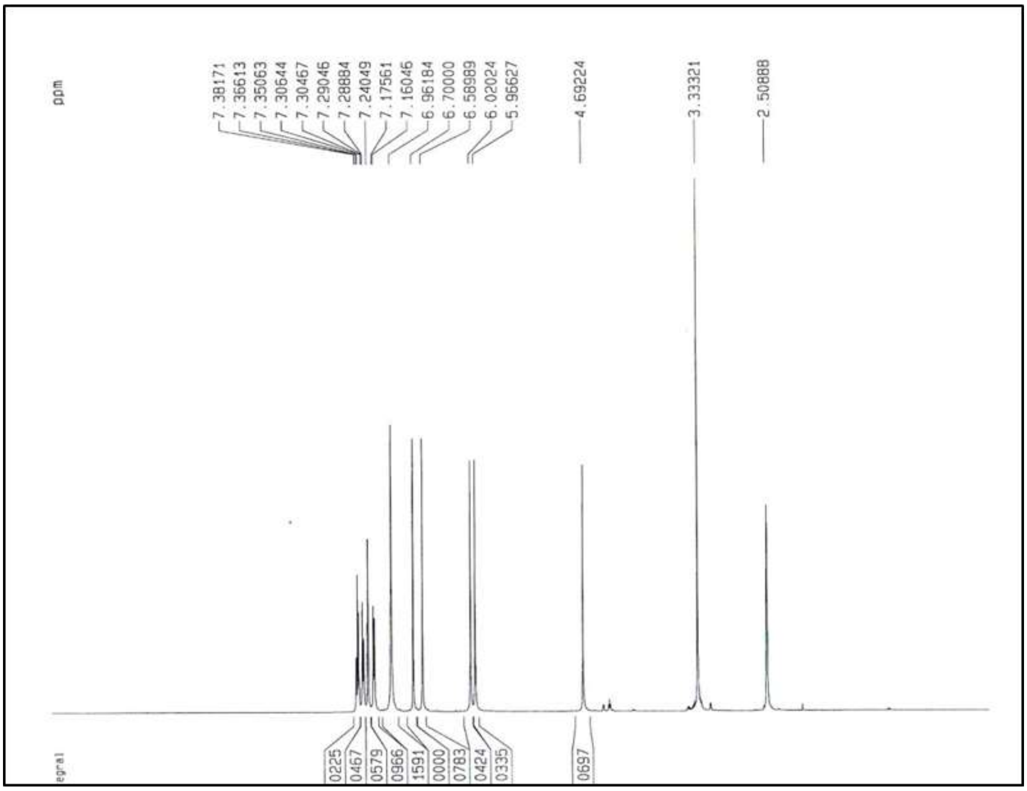

^{13}CNMR Spectrum: 6-Amino-8-(3-bromophenyl)-8H-[1,3]dioxolo[4,5-g]-chromene-7-carbonitrile, TABLE 3, Entry 6:

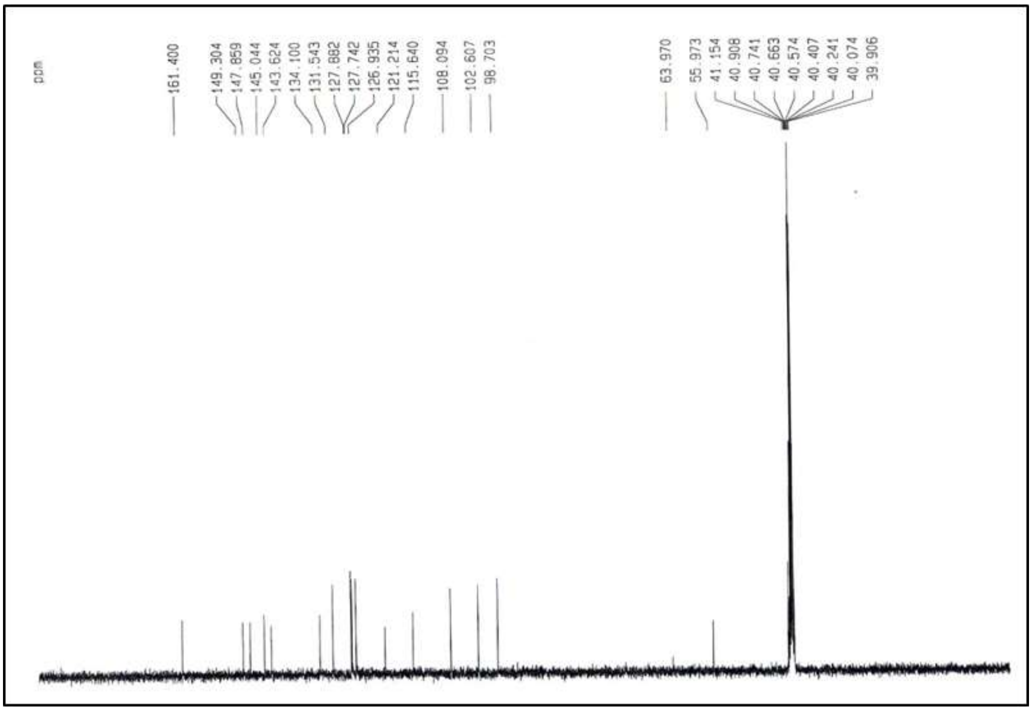

IR Spectrum: 2-Amino-4-(3-nitrophenyl)-3-cyano-4H,5H-pyrano[3,2-c]chromene-5-one, TABLE 4, Entry 8:

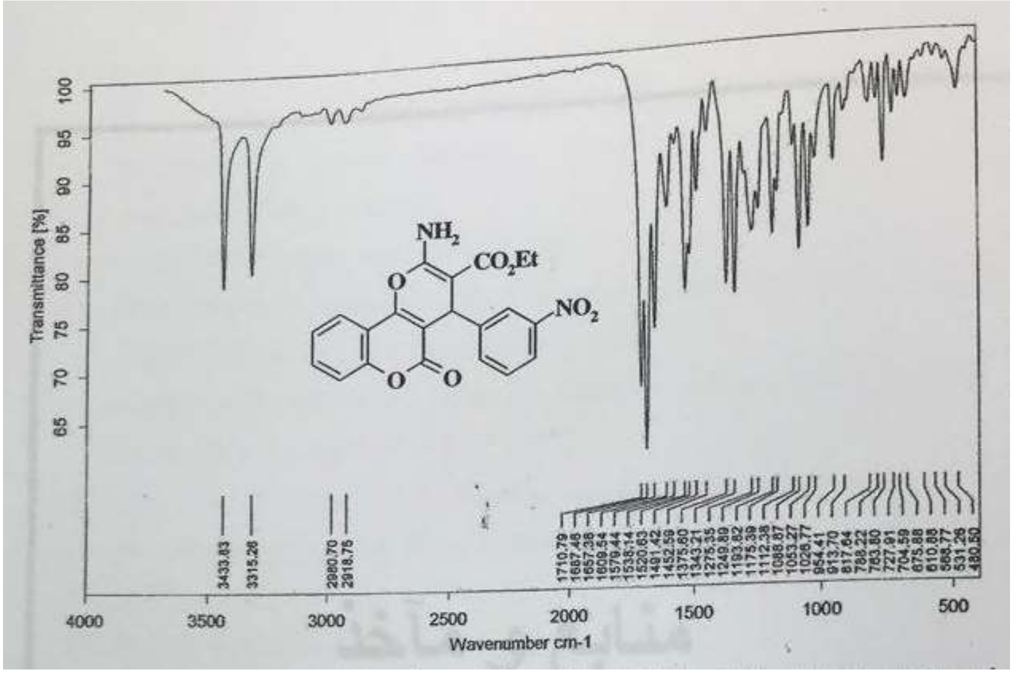

^{1}HNMR Spectrum: 2-Amino-4-(3-nitrophenyl)-3-cyano-4H,5H-pyrano[3,2-c]chromene-5-one, TABLE 4, Entry 8:

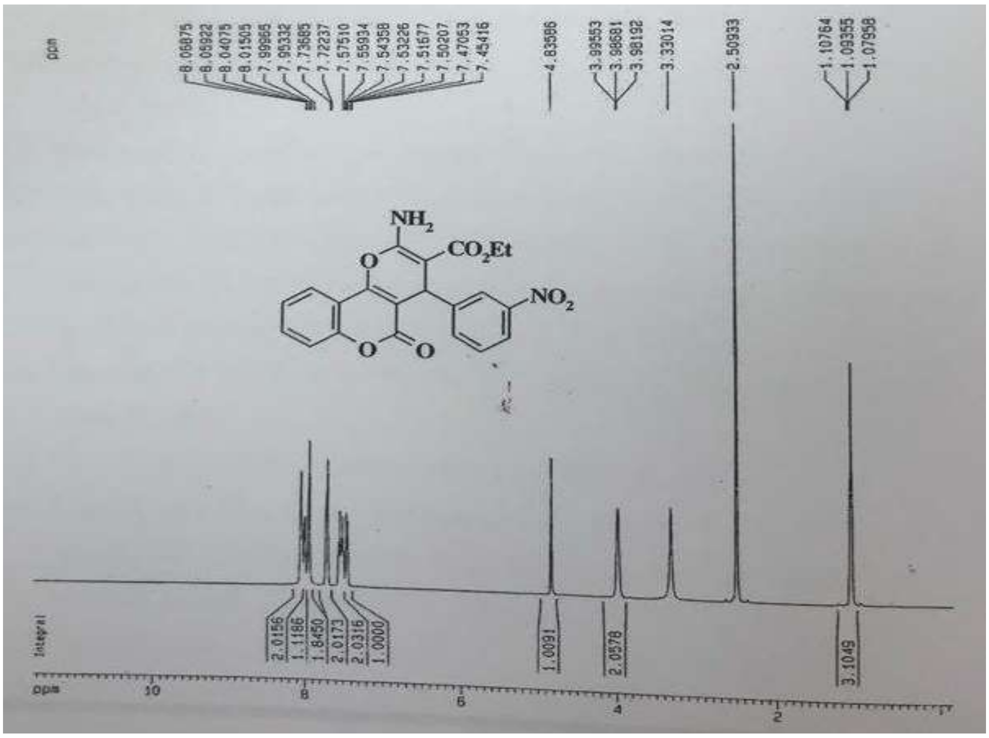

IR Spectrum: 2-Amino-4-(2,4-dichlorophenyl)-3-cyano-4H,5H-pyrano[3,2-c]chromene-5-one, TABLE 4, Entry 10:

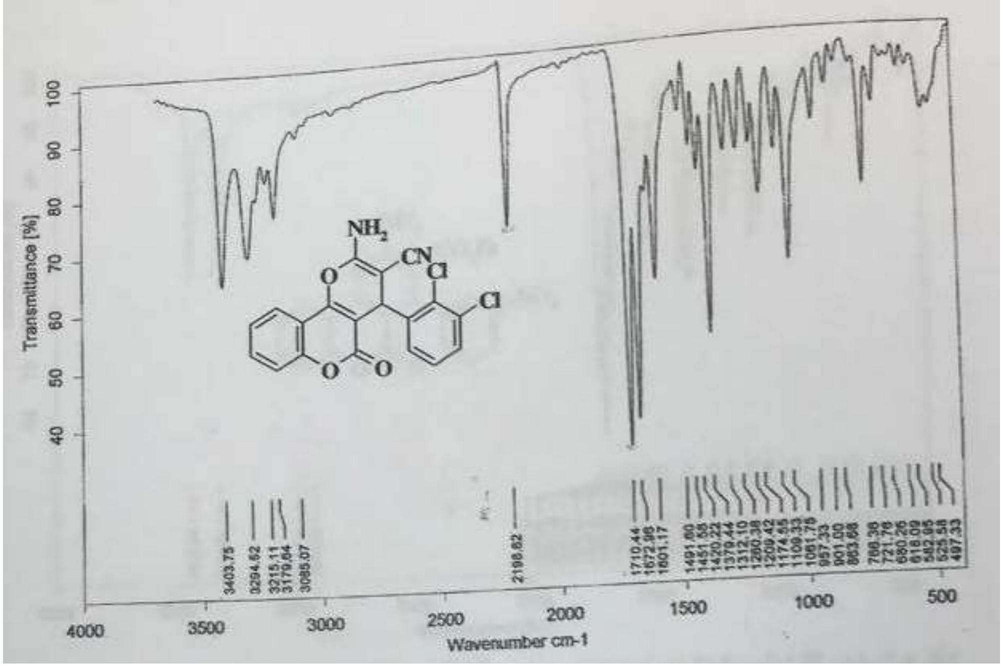

^{1}HNMR Spectrum: 2-Amino-4-(2,4-dichlorophenyl)-3-cyano-4H,5H-pyrano[3,2-c]chromene-5-one, TABLE 4, Entry 13:

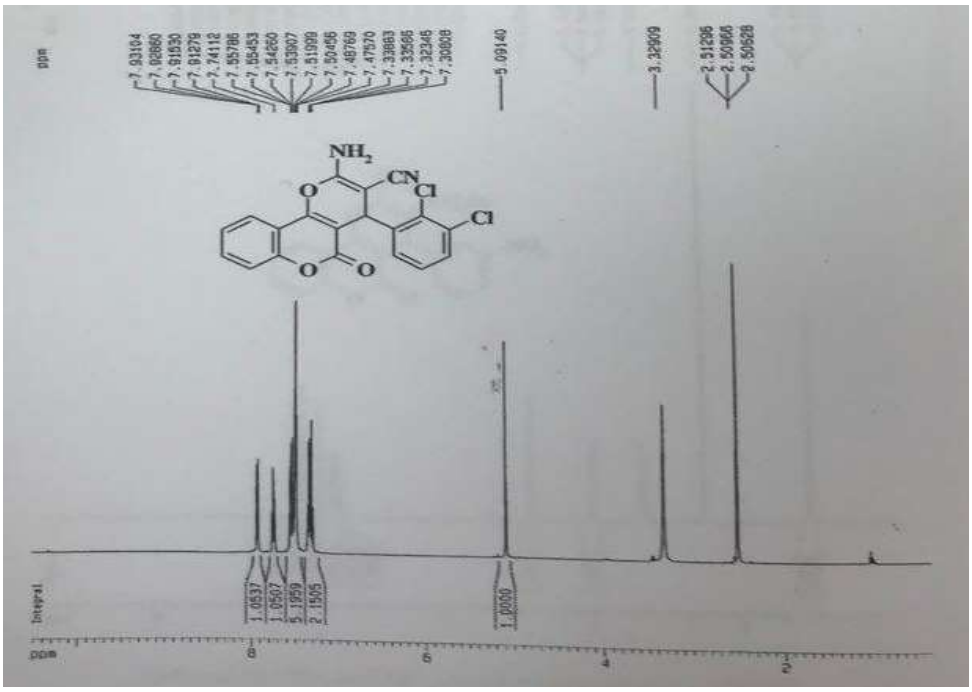

IR Spectrum: 6-Amino-5-cyano-3-methyl-1,4-diphenyl-1,4-dihydro-pyrano[2,3-c]pyrazole., TABLE 4, Entry 13:

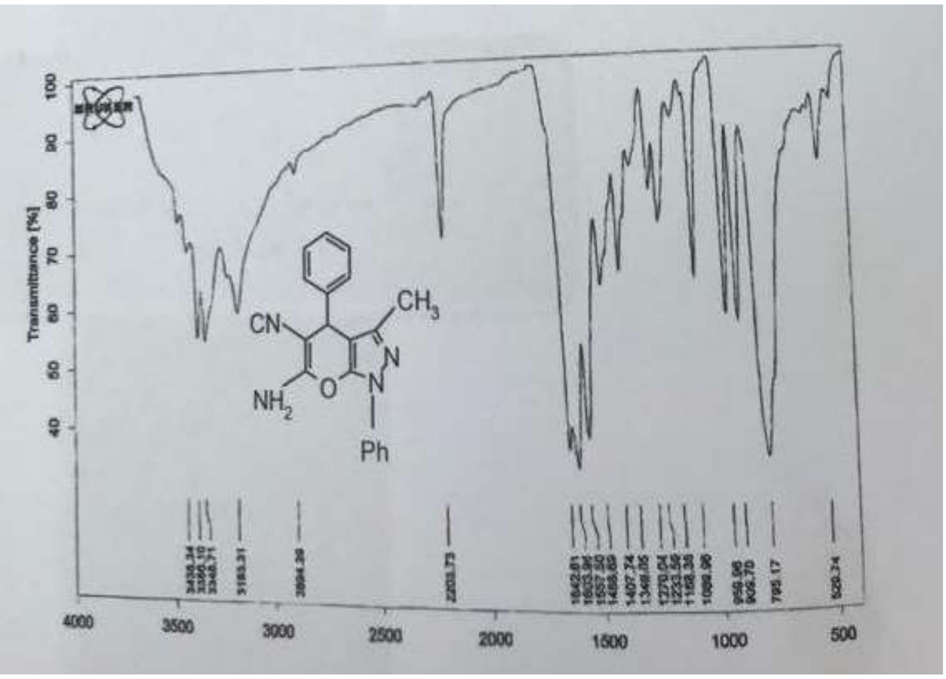

Supplement: Supplementary file 1 — Supplementary Information. [file 41598_2022_21196_MOESM1_ESM.pdf]
